# Systemic Barium Toxicity Manifesting As Acute Hypokalemic Paralysis and Respiratory Failure Following a Firework Injury

**DOI:** 10.7759/cureus.106268

**Published:** 2026-04-01

**Authors:** Nicholas L Todd, Michael Todd, Ji Young Chung, Alexander E Isla, Nicole Griffin, Jacob Roe, Shahriar Tahvilian, Frederick Korpi, Jeffrey Cochran, Mark Rose

**Affiliations:** 1 Orthopedic Surgery, Aultman Hospital, Canton, USA; 2 Neurology, University of Minnesota, Minneapolis, USA

**Keywords:** barium, firework, hypokalemia, paralysis, trauma

## Abstract

We present the case of a 63-year-old male who sustained a penetrating soft tissue injury to the right thigh from a commercial firework. Following uncomplicated surgical debridement and discharge, the patient returned within hours exhibiting rapidly progressive ascending paralysis, bulbar weakness, and respiratory failure requiring intubation. Laboratory evaluation revealed profound hypokalemia (1.4 mmol/L), hypophosphatemia, and rhabdomyolysis. The clinical presentation, coupled with the patient’s report of the firework emitting a green flare, is most consistent with acute barium toxicity. Formal toxicologic confirmation was not available; however, the clinical constellation, mechanism of injury, and rapid response to electrolyte repletion strongly support this diagnosis. Barium salts, commonly used in pyrotechnics to produce green coloration, can induce systemic toxicity by competitively blocking potassium channels, causing a widespread intracellular shift of potassium. This case highlights the rare but life-threatening systemic toxicity associated with soluble barium salts and the importance of considering toxicologic etiologies in trauma patients presenting with unexplained neurological collapse.

## Introduction

Firework-related injuries are typically mechanical or thermal, resulting in burns, blast injuries, or soft tissue trauma. However, pyrotechnic devices contain various metallic salts that produce specific colors and can pose significant toxicological risks. Barium salts, specifically barium chloride and barium nitrate, are frequently used to create green visual effects [1]. While insoluble barium sulfate is safely utilized in medical imaging, soluble barium salts are highly toxic. Upon systemic absorption, free barium ions act as potent competitive blockers of inward rectifier potassium channels, precipitating a rapid intracellular shift of potassium [2]. This results in profound hypokalemia and neuromuscular blockade. We describe a rare case of profound hypokalemic paralysis masquerading as acute neuromuscular disease following a penetrating firework injury, emphasizing the need for rapid recognition and aggressive electrolyte repletion.

## Case presentation

**Figure 1:**
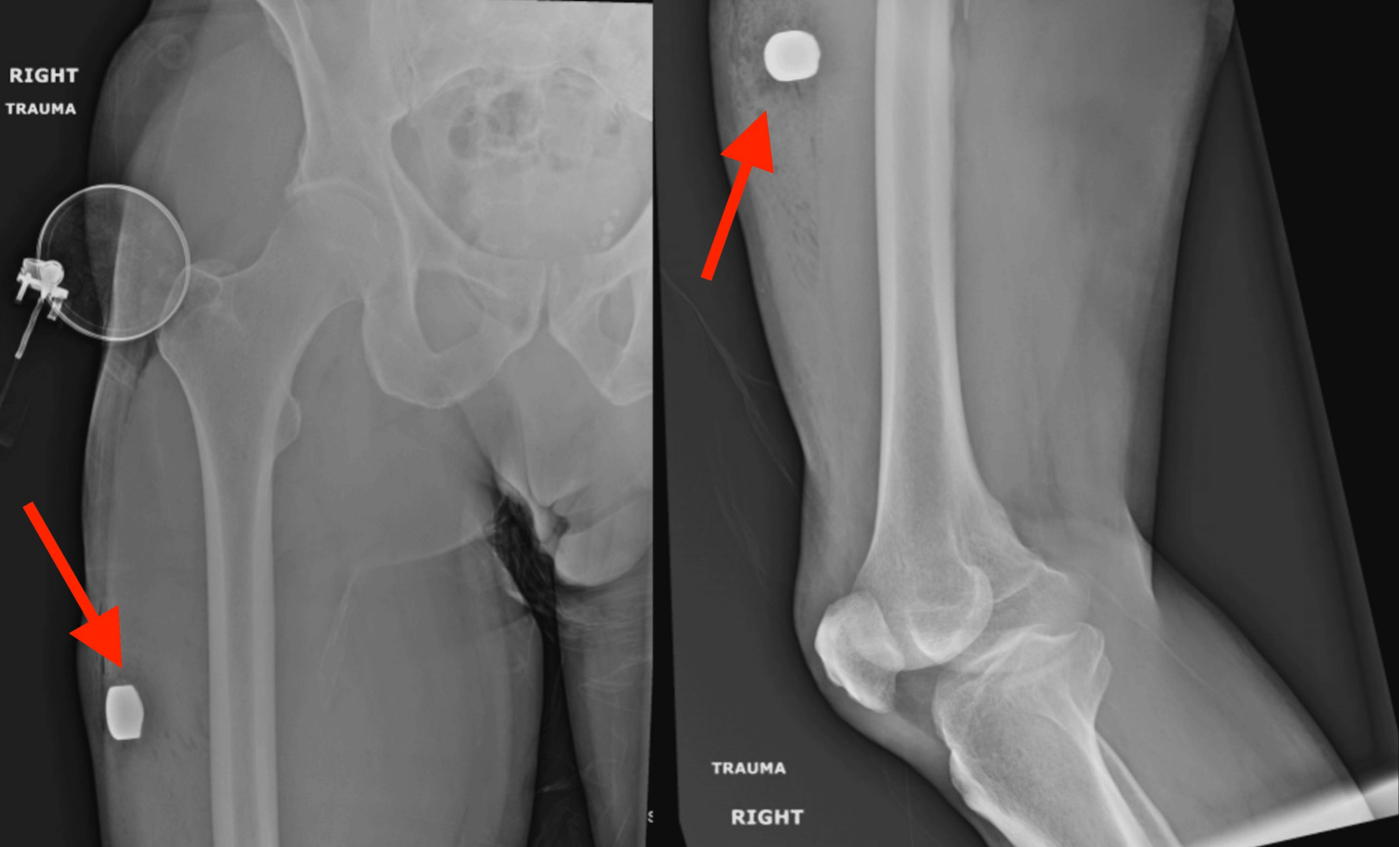
Anterior-posterior (AP) and lateral images of the right femur demonstrating a radiopaque foreign body in the anterolateral soft tissues of the thigh, as indicated by the red arrows.

A 63-year-old male presented to the ED as a trauma activation after being struck in the right thigh by a projectile from a firework approximately 50 feet away (Figure 1). Primary survey revealed a stable airway and hemodynamics. Examination of the right lower extremity demonstrated a penetrating wound to the anterior mid-thigh with a surrounding palpable hematoma. CT angiography confirmed an intramuscular radiopaque foreign body with an adjacent hematoma and excluded large-vessel arterial injury. The patient was admitted to the Orthopedic service and underwent irrigation and debridement (I&D) on hospital day 1. Intraoperative findings noted a circular foreign body (2 cm by 0.5 cm), which was removed intact. He was monitored uneventfully in the post-anesthesia care unit (PACU) and was discharged home later the same day after meeting mobilization goals with the physical therapy team, without postoperative restrictions.

Approximately four hours post-discharge, the patient returned to the ED with acute onset of generalized weakness, vomiting, and lightheadedness. The weakness began in the lower extremities and rapidly ascended, leading to an inability to ambulate. By the time of ED arrival, he reported worsening dysphagia and dysarthria. On physical examination, the patient was anxious and diaphoretic. Vital signs were significant for hypertension (175/96) and tachypnea with increased work of breathing. Neurological assessment revealed flaccid quadriplegia, with strength grading of 1/5 in the lower extremities and 2/5 in the upper extremities. Cranial nerve examination demonstrated dysarthria and dysphagia without facial weakness or ophthalmoplegia. Deep tendon reflexes were diffusely absent (0/4). Sensation remained fully intact to light touch in all four extremities, and the patient was alert and oriented, arguing against central nervous system depression. There were no asymmetric findings or sensory level to suggest spinal cord or brainstem pathology. Due to the rapid progression of bulbar symptoms, impending respiratory muscle fatigue, and threatened airway, the patient underwent rapid sequence intubation (RSI) and was admitted to the Surgical ICU.

Initial laboratory evaluation revealed critical electrolyte derangements and markers of muscle injury. Serum potassium was critically low at 1.4 mmol/L, and creatine phosphokinase (CPK) was markedly elevated at 1,559 U/L, consistent with rhabdomyolysis. Serum phosphorus was low at 1.7 mg/dL, and serum glucose was elevated at 219 mg/dL. A complete blood count showed leukocytosis at 17.2 k/mm3 with a neutrophilic predominance, likely reactive to surgery and stress. An arterial blood gas and coagulation studies were not documented. Serum and urine barium levels were not obtained; confirmatory toxicologic testing was not available at our institution during the acute presentation. Pertinent admission laboratory values are summarized in Table 1.

**Table 1:** Laboratory values on admission.

Analyte	Patient value	Reference range
Potassium	1.4 mmol/L	3.5-5.1 mmol/L
Phosphorus	1.7 mg/dL	2.5-4.5 mg/dL
Magnesium	1.8 mg/dL	1.6-2.6 mg/dL
Creatine phosphokinase (CPK)	1559 U/L	39-308 U/L
Glucose	219 mg/dL	74-106 mg/dL
WBC count	17.2 × 10³/µL	4.5-11.0 × 10³/µL
Lactic acid	1.4 mmol/L	0.5-2.0 mmol/L

A CT of the head and cervical spine showed no acute intracranial or spinal pathology. The patient’s history was reviewed, and he specifically noted that the firework had flared green before striking him, raising high suspicion for barium toxicity given the clinical constellation of a “green firework” injury and severe hypokalemia.

The patient was managed with aggressive electrolyte replacement to counteract the intracellular potassium shift. All potassium was administered intravenously via central venous access, with continuous cardiac telemetry monitoring throughout. Replacement was initiated within one hour of admission to the Surgical ICU and titrated to serial serum potassium levels obtained every two to four hours. Magnesium sulfate supplementation was not additionally required given a borderline-normal serum magnesium of 1.8 mg/dL; however, providers considered its use given its role in potassium retention. Sodium sulfate administration was not pursued, as this antidote is generally reserved for gastrointestinal decontamination in oral ingestions and would not be expected to benefit a wound-entry exposure. Rebound hyperkalemia was monitored closely once serum potassium normalized, as resolution of barium blockade can cause rapid return of intracellular potassium to the serum. The total electrolyte replacement administered over the first 24 hours is detailed in Table 2.

**Table 2:** Total amount of each electrolyte replenished during admission.

Electrolyte / Medication	Quantity administered
Potassium bicarbonate	130 mEq
Potassium chloride	170 mEq
Potassium phosphate	40 mmol

By the morning of hospital day 2, serum potassium had normalized to 5.2 mmol/L. The patient self-extubated and demonstrated dramatic neurological recovery. Examination revealed 5/5 strength in the upper and lower extremities. Neurology was consulted and deferred MRI imaging given the rapid resolution of symptoms coinciding with electrolyte correction. The patient was discharged on hospital day 4 with a diagnosis of heavy metal toxicity secondary to firework injury. He followed up for his two-week postoperative visit in the orthopedic clinic with no signs of weakness and a healed surgical wound.

## Discussion

This case illustrates the classic toxidrome of soluble barium salts, a rare presentation in the context of trauma. The mechanism of toxicity involves competitive blockade of inward rectifier potassium channels. Under normal physiology, these channels allow potassium to exit the cell to help maintain the resting membrane potential. Barium ions block this efflux while the sodium-potassium ATPase pump continues to transport potassium into the cell [3].

This “trapping” mechanism results in a massive shift of potassium from the extracellular to the intracellular space, leading to profound serum hypokalemia without a total-body potassium deficit [4]. The resulting hyperpolarization of the cell membrane renders skeletal muscle unexcitable, causing flaccid paralysis and areflexia. The preserved sensation and consciousness seen in this patient are typical, as barium affects skeletal muscle channels more significantly than neuronal conduction [2].

The differential diagnosis for acute ascending paralysis typically includes Guillain-Barré syndrome (GBS) and periodic paralyses. However, the timeline in this case was too rapid for typical GBS, which usually progresses over days to weeks, whereas this patient decompensated to respiratory failure in under six hours. Familial hypokalemic periodic paralysis is rare in this age group without a prior history. The temporal relationship between the injury from a green pyrotechnic device, known to contain barium salts such as barium nitrate or chlorate, and the onset of symptoms, combined with the profound hypokalemia (1.4 mmol/L), preserved sensation and consciousness, areflexia, and rapid reversal following aggressive potassium repletion, makes barium toxicity the most probable diagnosis [5]. The absence of confirmatory serum or urine barium levels should be acknowledged; however, formal toxicologic testing is frequently unavailable in the acute emergency setting, and the clinical and physiologic criteria are sufficient for presumptive diagnosis and targeted management.

Treatment is primarily supportive, focusing on airway management and aggressive IV potassium repletion. Confirmatory testing for barium toxicity may include serum barium levels, urine barium quantification, or consultation with a medical toxicologist or regional poison control center; however, these resources are frequently unavailable in acute emergency settings, and clinical diagnosis must not be delayed while awaiting confirmatory results [4,6]. While some literature suggests the use of sodium sulfate to precipitate barium into insoluble barium sulfate, this is generally reserved for oral ingestions to reduce gastrointestinal absorption [4]. In cases of systemic absorption via wound entry, aggressive potassium replacement is the mainstay of therapy to overcome competitive blockade at the inward rectifier potassium channel. In this case, the profound hypokalemia (1.4 mmol/L), flaccid areflexic quadriplegia with preserved sensation, and rapid neurological recovery paralleling potassium normalization to 5.2 mmol/L all align closely with the known toxidrome of soluble barium salts. Providers must maintain vigilance for rebound hyperkalemia once the barium blockade resolves and intracellular potassium shifts back into the serum; continuous cardiac monitoring is therefore essential throughout the repletion period [5].

## Conclusions

Medical providers should maintain a high index of suspicion for chemical toxicity in patients with pyrotechnic injuries, particularly when the clinical course involves unexplained neurological or electrolyte abnormalities. The presence of a green firework in the history is a specific red flag for barium exposure. Early recognition allows for targeted supportive care and may prevent unnecessary neurological workups for demyelinating disorders.
